# Total buried bumper syndrome: A case study in transabdominal removal using a technique of endoscopic submucosal dissection

**DOI:** 10.1002/ccr3.2409

**Published:** 2019-09-03

**Authors:** Hajime Nakamura, Shohei Kikuchi, Hiroyuki Ohnuma, Masahiro Hirakawa, Junji Kato

**Affiliations:** ^1^ Department of Medical Oncology Sapporo Medical University School of Medicine Sapporo Japan

**Keywords:** buried bumper syndrome, endoscopic submucosal dissection, percutaneous endoscopic gastrostomy

## Abstract

Tube removal by endoscopic submucosal dissection using needle and insulation‐tipped diathermic knives against buried bumper syndrome is a reliable, noninvasive and safe procedure.

Buried bumper syndrome (BBS) is a rare but well‐recognized complication of percutaneous endoscopic gastrostomy (PEG).^1^ Endoscopic removal is often attempted but can result in failure or complications. Surgical removal is reliable but invasive.

A 74‐year‐old female underwent a PEG with a bumper‐button type device (Figure 1). BBS was endoscopically diagnosed since the inner bumper was totally covered by gastric mucosa (Figure 2). A mucosal incision and submucosal dissection using needle and insulation‐tipped diathermic knives for the bumper‐covering mucosa were performed. The PEG tube was transabdominally removed through the fistula without complications (Video S1).

**Figure 1:**
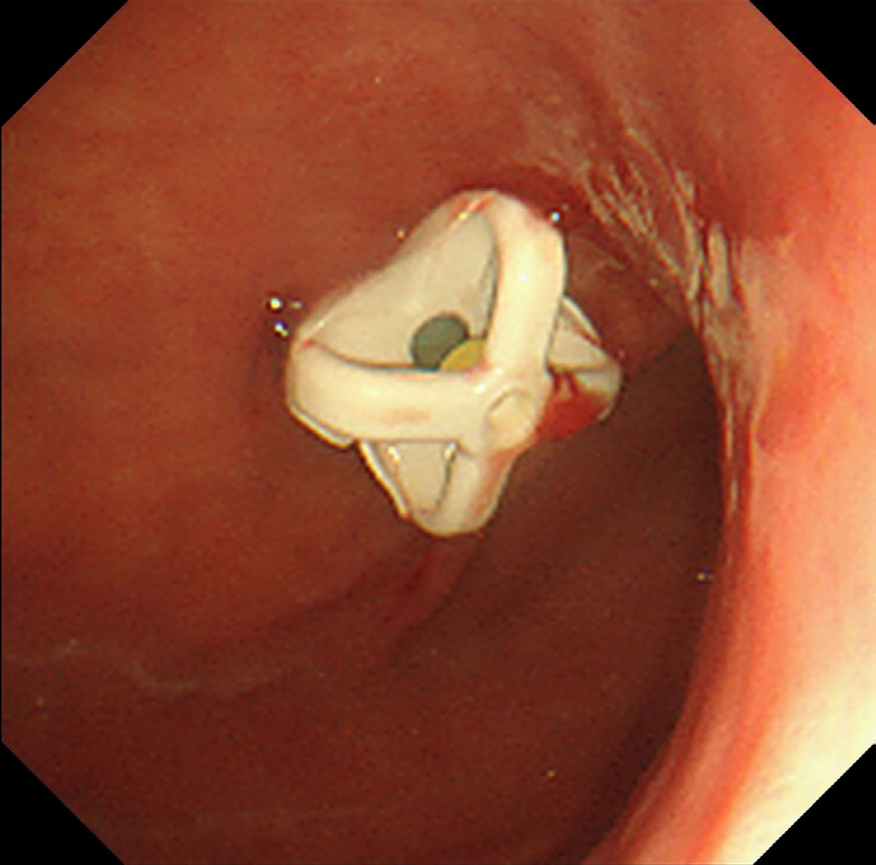
Percutaneous endoscopic gastrostomy (PEG) with a bumper‐button type device was performed through the anterior wall of the lower body of the stomach

**Figure 2:**
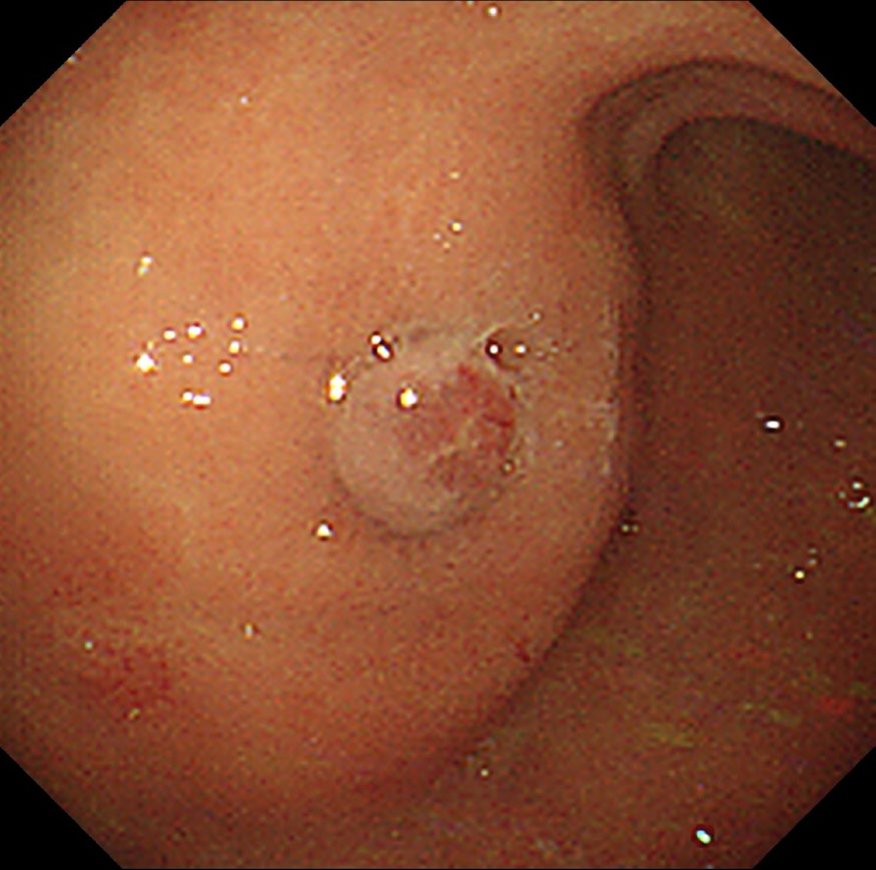
An upper gastrointestinal endoscopy performed for the purpose of tube removal revealed the inner bumper was totally covered by gastric mucosa

## CONFLICT OF INTEREST

None declared.

## AUTHOR CONTRIBUTIONS

HN and SK drafted the manuscript. HO and MH involved in the procedure. JK performed a critical review.
